# Editorial: Employee resilience, volume II

**DOI:** 10.3389/fpsyg.2026.1838808

**Published:** 2026-04-15

**Authors:** José Alberto Martínez-González, Carmen D. Álvarez-Albelo, Urszula Kobylińska, Almudena Barrientos-Báez

**Affiliations:** 1 Departamento de Dirección de Empresas e Historia Económica, Cátedra de Turismo CajaCanarias-Ashotel-ULL, Cátedra CEOE-ULL, Instituto Universitario de Desarrollo Regional and Instituto Universitario de la Empresa, Universidad de La Laguna, Canary Islands, Spain; 2 Departamento de Economía, Contabilidad y Finanzas, Cátedra de Turismo CajaCanarias-Ashotel-ULL, Instituto Universitario de Investigación Social y Turismo and Instituto Universitario de la Empresa, Universidad de La Laguna, Canary Islands, Spain; 3 Faculty of Engineering Management, Bialystok University of Technology, Bialystok, Poland; 4 Departamento de Teorías y Análisis de la Comunicación, Facultad de Ciencias de la Información, Universidad Complutense de Madrid, Madrid, Spain

**Keywords:** employee resilience, determinants, interventions, Research Topic, volume ii

Employee resilience is defined as the ability to adapt to workplace turbulence and demands while maintaining effective performance by utilizing personal and contextual resources (Britt et al., 2016; Hartmann et al., 2020). It is a dynamic capacity that responds to daily stressors and acts as a mediator between working conditions and job performance (Zhu et al., 2025). Due to its effect on employees' wellbeing and organizations' capacity to adapt to volatile and uncertain contexts, such as those encountered today, identifying and comprehending the factors that develop or diminish resilience is a highly relevant area of research (King et al., 2016; Hartmann et al., 2020). Furthermore, though resilience can be developed, interventions to strengthen it must be well-designed and targeted at specific risk profiles, highlighting the importance of elucidating its determinants.

The studies compiled in this volume contribute significantly to our understanding of these determinants, as well as to interventions to promote employee resilience. The works are organized around three major themes identified in the literature (Galy et al., 2023): individual emotional and cognitive mechanisms; team dynamics and leadership; and organizational conditions.

Within the first theme, Zeng et al.'s study of nurses in Chinese tertiary hospitals analyzes how different patterns of emotional labor—surface acting-suppression, deep acting, and natural engagement—are associated with varying levels of psychological resilience. The authors find that surface acting-suppression is associated with a higher level of resilience, followed by deep acting and then natural engagement. Therefore, resilience is not distributed uniformly among nurses but varies depending on the psychological resources each individual is able to mobilize. Based on these results, the authors propose intervention measures that could be useful in this work context. The work of Liu et al. investigates the effects of time mental accounting on intertemporal decision-making among young workers, and whether these effects operate via time management disposition (TMD) and future self-continuity (FSC). It is found that mental budgeting and loss aversion are positively associated with TMD and FSC. Furthermore, TMD and FSC mediate the effects of time mental accounting on intertemporal choices. In light of these results, exercises of time budgeting, future self-visualization, mentoring, goal review, and time-management training could improve young workers' strategic planning capabilities.

Turning to the second thematic area, Li and Li explore the cognitive and affective mechanisms through which authentic leadership strengthens resilience. The authors identify role breadth self-efficacy and employee vigor as key mediators within the framework of Cognitive-Affective Theory of Personality. However, employee traditionalism plays a negative moderating role between authentic leadership and employee vigor. These results highlight the importance of recruiting leaders who exhibit positive and authentic behavior, prioritizing the emotional and cognitive wellbeing of their workers, and taking their level of traditionalism into account when designing measures to foster resilience. Piao and Hahn examine the relationship between safety leadership and employee safety behaviors in China's electricity industry. Their findings confirm that such leadership positively influences safety compliance and participation, with employees' safety knowledge mediating this relationship. Furthermore, the positive effect of safety knowledge on compliance and participation is greater among employees with higher psychological resilience. Therefore, the importance of investing in safety leadership training and implementing interventions to increase workers' resilience is evident. Drawing on Cognitive-Affective Systems Theory, Um et al. explore the relationship between coaching leadership and incremental innovation and employee performance. They investigate the mediating roles of psychological safety (cognitive factor) and positive group affect (emotional factor) in this relationship. Their analysis shows that coaching leadership fosters both incremental innovation and positive group affect. Furthermore, positive group affect links coaching leadership to employee outcomes, while the influence of psychological safety depends on the context. These findings can guide organizations in promoting innovation and workforce performance. Conversely, Li and Hahn demonstrate that narcissistic leadership weakens employee resilience and inhibits innovative behavior in SMEs, with employee resilience acting as a mediating factor in this relationship. They also found that a supportive team environment moderates these negative effects. Therefore, a supportive work environment is important in protecting against the effects of negative leadership and preserving employee resilience and their capacity for innovation.

In the third theme, Renk and Sutter review the literature on the relationship between work-related extended availability, work-family conflict and health outcomes. The reviewed studies show that increased availability is associated with greater work-family conflict and poorer health outcomes. The literature has also identified moderating factors, such as control over workload, greater worker autonomy, and a management style that respects the boundaries between work and family life. Nakamura et al. developed a valid and reliable loneliness scale for the workplace. Their analysis revealed a four-factor structure comprising sense of loneliness, sense of usefulness, emotional connections, and sense of support. Sense of usefulness is a novel addition compared to other scales in the literature. This scale is a valuable tool for designing interventions aimed at reducing stress and promoting employee wellbeing. In accordance with the Job Demands-Resources model, Scurtu-Tura et al. question the usefulness of generational labels for predicting work engagement in Spain. Specifically, they found that sociodemographic variables such as marital and employment status were stronger predictors of work engagement. This suggests that organizations' HR strategies should prioritize life stage and resource management over generational stereotypes. Consistent with this model, Geisler et al.'s study of municipal social welfare services in Sweden reveals that actively seeking challenges enhances the positive impact of supervisor support on job satisfaction. Furthermore, job crafting aimed at reducing demands does not offset the negative impact of overload or role conflict. The authors interpret these results as evidence that the organizational context greatly limits employees' ability to proactively redesign their work. Therefore, organizations must provide the necessary resources, autonomy and managerial support for such behavior. Finally, the bibliometric study by Ramzuni et al. provides an overview of 25 years of research on employee agility, a concept closely associated with adaptability and resilience in the face of change. Their results reveal an evolution from an HR management–centered perspective to a multidimensional view that integrates psychological, organizational, and socio-technological factors. They also highlight some issues addressed in this volume as areas for future research: the role of leadership, the relationship with innovation, and the underrepresentation of the public sector.

These findings provide a solid foundation for further understanding of this complex phenomenon, whose relevance is paramount for workers, organizations and academics in today's uncertain times.
